# Sarcopenia in hospitalized geriatric patients: insights into prevalence and associated parameters using new EWGSOP2 guidelines

**DOI:** 10.1038/s41430-020-00780-7

**Published:** 2020-10-15

**Authors:** Dominic Bertschi, Caroline M. Kiss, Nadine Beerli, Reto W. Kressig

**Affiliations:** 1 University Department of Geriatric Medicine FELIX PLATTER, Basel, Switzerland; 2 Department of Geriatrics, Bern University Hospital, and University of Bern, Bern, Switzerland

**Keywords:** Epidemiology, Skeletal muscle

## Abstract

**Background:**

Data on prevalence of sarcopenia and its associated parameters in hospitalized geriatric patients are heterogeneous due to various definitions of the disease. The aim of this study was to determine the prevalence of sarcopenia using latest recommendations of the European Working Group on Sarcopenia in Older People 2 (EWGSOP2), and to investigate associated parameters in patients admitted to acute geriatrics and geriatric rehabilitation.

**Methods:**

In this cross-sectional single-centre study including 305 hospitalized geriatric patients, handgrip strength (pneumatic hand dynamometer) and muscle quantity (body impedance analysis) were assessed. Probable sarcopenia was defined by low handgrip strength, and the diagnosis was confirmed when both handgrip strength and muscle quantity were below cut-off points. Furthermore, parameters of the geriatric baseline examination were analyzed for association with probable and confirmed sarcopenia using logistic regression models.

**Results:**

Median age of the study population was 84.0 years, and 65.6% were female. The prevalence of probable sarcopenia was 24.6% (CI 19.8–29.4%), and the prevalence of confirmed sarcopenia was 22.6% (CI 17.9–27.3%). Low calf circumference, low body mass index, cognitive impairment and an increased risk of malnutrition were found to be associated with confirmed sarcopenia. In contrast, only cognitive impairment was positively associated with probable sarcopenia.

**Conclusions:**

Sarcopenia is highly prevalent in geriatric inpatients, and multiple parameters were found to be associated with the disease. To reduce negative clinical outcomes, our findings support the need for routinely performed admission examinations for prompt diagnosis of sarcopenia, and a timely start of treatment in hospitalized geriatric patients.

## Introduction

Sarcopenia, defined as loss of muscle mass and function [1], is a common condition in older individuals associated with various comorbidities such as malnutrition and cognitive impairment [2, 3]. It further was found to be associated with adverse clinical outcomes such as falls and fractures, functional decline and increased mortality [4–7].

In the last years, heterogeneous data on the prevalence of sarcopenia have been reported due to various operational definitions and cut-off points [8]. Recently, the European Working Group on Sarcopenia in Older People 2 (EWGSOP2) published an updated evidence-based consensus report on its definition and diagnosis. These new guidelines focus on low muscle strength as a key characteristic of sarcopenia, use low muscle mass to confirm the diagnosis, and suggest poor physical performance as an indicator for severe sarcopenia [9]. Criteria of EWGSOP2 have been used for different patient cohorts and settings. Studies reported prevalence rates of sarcopenia between 0.4 and 9.3% in community-dwelling older adults [7, 10–13]. Higher rates of prevalence have been found in other patient populations such as octogenarian males (20.0%) [14] and in patients with liver cirrhosis (28.2%) [15]. Nonetheless, data on the prevalence of sarcopenia in hospitalized geriatric patients are scarce [16–18]. So far, no study has investigated parameters associated with probable and confirmed sarcopenia using the revised diagnostic criteria of EWGSOP2.

The present study aimed to investigate the prevalence of sarcopenia and its associated parameters in patients admitted to acute geriatrics and geriatric rehabilitation, using the recommended diagnostic criteria of EWGSOP2. Given the prevalence rates of existing data between 18.1 and 22.8% [16–18], we hypothesized that the prevalence of sarcopenia is higher than 20.0% in both geriatric inpatient settings. Furthermore, we hypothesized that parameters from the geriatric baseline evaluation associated with probable and confirmed sarcopenia can be validated if diagnostic criteria of EWGSOP2 are used.

## Methods

### Study population

This cross-sectional study was performed at a university department of geriatric medicine between September 10 and October 30, 2019. A consecutive convenience sample of all patients older than 65 years, admitted to acute geriatrics and geriatric rehabilitation, was asked to participate. Patients with acute sepsis, severe dehydration or volume overload, life expectancy of <3 months according to attending physician, non-removable plasters or bandages at feet or hands aggravating body impedance analysis (BIA), implanted defibrillation device, and inability to follow study procedures (e.g., due to delirium or language barriers) were excluded. All participants signed an informed consent form before any study procedure was performed. If a patient was not capable of giving informed consent due to severe cognitive impairment, the consent form was signed by a proxy person. In addition to medical treatment, the standardized rehabilitation program during hospitalization included physiotherapy, occupational and nutritional therapy for a total of 300 min/week in acute geriatric patients, and 450 min/week for patients in geriatric rehabilitation.

The study was approved by the Ethics Committee of Northwest and Central Switzerland (registration ID 2019–01461) and was registered at ClinicalTrials.gov (NCT04124575).

### Geriatric baseline examination

For all included participants, age, sex, length of hospital stay, comorbidities and number of drugs at hospital admission were extracted from medical records. Body height (cm), weight (kg), calf and mid-arm circumference (cm) were measured using standard methods. Results of anthropometric measures and the geriatric assessment were dichotomized at following cut-off points: Body mass index (BMI) <22 kg/m^2^ for persons ≥70 years old and <20 kg/m^2^ for persons <70 years old (low BMI) [19]; calf circumference <31 cm (low calf circumference) [9]; mid-arm circumference (MAC) <26.5 cm for men and <24.5 cm for women ≥75 years old, and <27.5 cm for men and <25.5 cm for women <75 years old (low MAC) [20]; mini mental state exam (MMSE) <27 points (cognitive impairment) [21]; nutritional risk screening (NRS 2002) ≥3 points (at risk of malnutrition) [22, 23]; timed up and go test (TUG) ≥20 s (low physical performance) [24]. Patients who were not able to perform the TUG at admission were scored as mobility impaired. The functional independence measure (FIM) score was used to assess the functional status. This clinician-reported score ranges from 18 to 126 points with higher scores reflecting better functionality [25].

### Assessment of sarcopenia

All participants were assessed for sarcopenia within the first 6 days of admission according to the recently defined EWGSOP2 guidelines [9]. To determine muscle strength, handgrip strength (HGS) of the dominant hand was measured with a pneumatic hand dynamometer (Martin Vigorimeter^®^, Gebrüder Martin GmBH, Tuttlingen, Germany). HGS was measured three times and the highest value was used for analysis. To define low HGS, cut-off points of <50 kPa for men and <34 kPa for women >75 years old, and <64 kPa for men and <42 kPa for women ≤75 years old were applied [26]. BIA was performed with a tetrapolar whole-body BIA device (BIA 101, Akern, Florence, Italy) to determine muscle mass and phase angle, a measurement of cell integrity. Thereby, a low value represents a decreased cellular integrity or even cell death, whereas a high value represents a larger amount of intact cell membranes [27]. All participants were assessed in supine position with extremities stretched. The estimates obtained for the evaluation of appendicular skeletal muscle mass (ASMM) derive from proprietary manufacturer algorithms and were applied using Bodygram Plus software, version 1.2.2.8 (Akern, Florence, Italy). Cut-off points for low appendicular skeletal muscle mass index (ASMI), calculated from ASMM/height^2^, were <7 kg/m^2^ for men and <5.5 kg/m^2^ for women [9]. According to EWGSOP2 guidelines, sarcopenia was defined as probable when HGS was low; diagnosis was made when both HGS and muscle quantity were low, and sarcopenia was defined as severe by additional documentation of low physical performance (TUG ≥20 s).

### Statistical analysis

The study population was characterized using frequencies (*n*) and percentages (%) for categorical variables, and medians and 25th to 75th interquartile ranges (IQR) for continuous data. Patient characteristics in acute geriatrics and geriatric rehabilitation were analyzed using Pearson–Chi-square test, Fisher’s exact test and Mann–Whitney *U* test, where appropriate. For power analysis, we used the method described by Daniel et al. to calculate the sample size for cross-sectional studies [28]. According to previous reports, we assumed a prevalence of sarcopenia of 25% [16–18]. Using a level of confidence of 95% and a precision of 5%, the calculated minimal sample size was *n* = 289 [29]. Prevalence of sarcopenia was reported as percentage (%) and 95% confidence interval (CI). In patients assessed for sarcopenia in acute geriatrics as well as geriatric rehabilitation, the Cohen’s kappa coefficient was calculated to test the reliability between the first and the second assessment for sensitivity analysis [30]. A multiple logistic regression model including the parameters age, sex, low calf circumference, low MAC, low BMI, cognitive impairment, low physical performance, risk of malnutrition and functional disability was applied to assess the association of different parameters with probable and confirmed sarcopenia. *P* values <0.05 were considered to be statistically significant. All statistical analyses were performed using SPSS Statistics, Version 22 (IBM SPSS Statistics, Chicago, IL).

## Results

Out of 414 patients admitted to acute geriatrics and geriatric rehabilitation, 29 were excluded because of acute sepsis, severe dehydration or volume overload. Thirteen patients had a remaining life expectancy of <3 months, two patients had an implanted defibrillation device, 12 patients refused informed consent, and 16 patients were excluded for other reasons, e.g., plasters or bandages at feet or hands that could not be removed. Overall, 342 admitted patients were assessed for sarcopenia. Out of these, we excluded the second measurement of 37 participants, as they were assessed twice due to their admission to acute geriatrics prior to rehabilitation, resulting in a final study population of 305 patients. Acute illnesses representing the main cause of hospitalization were orthopaedic (38.0%), neurological (28.2%), infectious (15.4%) and cardiovascular (9.2%) diseases. In addition, 9.2% of the participants were hospitalized for other diseases.

### Clinical characteristics of the study population

Baseline characteristics of the study population, stratified by wards (acute geriatrics and geriatric rehabilitation) and by gender (males and females), are presented in Table 1. The median (interquartile range) age of the study population was 84.0 (10.0) years, and 65.6% of our study participants were female. Patients admitted to acute geriatrics had a significant higher number of comorbidities compared to patients admitted to geriatric rehabilitation (5.0 and 4.0, respectively, *p* = 0.002) and were more often cognitively impaired (67.9% and 55.4%, respectively, *p* = 0.029). Overall, male patients were more often bedridden (35.2% and 24.0%, respectively, *p* = 0.038), suffered more often from a coronary heart disease (42.9% and 29.0%, respectively, *p* = 0.015), and demonstrated a lower level of functionality than female patients (FIM score 70.0 points and 78.5 points, respectively, *p* = 0.007), whereas females were significantly more osteoporotic than males (32.5% and 19.0%, respectively, *p* = 0.013).

**Table 1:** Characteristics of the study population, stratified by wards (acute geriatrics and geriatric rehabilitation) and by gender (males and females).

Characteristic	All *n* = 305	Acute geriatrics *n* = 193	Geriatric rehabilitation *n* = 112	Males *n* = 105	Females *n* = 200
General characteristics					
Age, years, median (IQR)	84.0 (10.0)	84.0 (10.0)	84.0 (8.8)	84.0 (11.5)	84.0 (10.0)
BMI, kg/m^2^, median (IQR)	25.6 (6.6)	25.5 (6.4)	25.6 (6.1)	26.0 (5.0)	25.2 (7.2)
Calf circumference, cm, median (IQR)	32.5 (5.0)	32.0 (5.0)	33.5 (5.3)	33.0 (4.0)	32.0 (5.4)
Mid-arm circumference, cm, median (IQR)	26.5 (5.5)	26.0 (5.0)	26.5 (6.4)	27.0 (4.5)	26.0 (5.9)
Phase angle, degree, median (IQR)	4.6 (0.8)	4.6 (0.7)	4.5 (0.8)	4.7 (1.0)	4.5 (0.8)
Bedridden, *n* (%)	85 (27.9)	55 (28.5)	30 (26.8)	37 (35.2)	48 (24.0)^a^
Length of hospital stay, days, median (IQR)	16.0 (9.0)	16.0 (9.0)	21.0 (12.8)^a^	17.0 (12.0)	16.0 (9.0)
Comorbidities					
Hypertension, *n* (%)	211 (69.2)	135 (69.9)	76 (67.9)	73 (69.5)	138 (69.0)
Coronary heart disease, *n* (%)	103 (33.8)	63 (32.6)	40 (35.7)	45 (42.9)	58 (29.0)^a^
Chronic kidney disease, *n* (%)	138 (45.2)	92 (47.7)	46 (41.1)	53 (50.5)	85 (42.5)
Diabetes, *n* (%)	74 (24.3)	47 (24.4)	27 (24.1)	32 (30.5)	42 (21.0)
Osteoporosis, *n* (%)	85 (27.9)	56 (29.0)	29 (25.9)	20 (19.0)	65 (32.5)^a^
Number of drugs, median (IQR)	7.0 (5.0)	7.0 (5.0)	7.0 (4.0)	7.0 (4.0)	7.0 (5.0)
Number of comorbidities, median (IQR)	5.0 (2.0)	5.0 (2.0)	4.0 (3.0)^a^	5.0 (2.0)	5.0 (2.0)
Geriatric assessment parameters					
Cognitive Impairment (MMSE <27 points), *n* (%)	193 (63.3)	131 (67.9)	62 (55.4)^a^	70 (66.7)	123 (61.5)
At risk of malnutrition (NRS ≥3 points), *n* (%)	165 (54.1)	100 (51.8)	65 (58.0)	58 (55.2)	107 (53.5)
FIM score, points, median (IQR)	76.0 (28.0)	75.0 (28.5)	78.5 (26.8)	70.0 (29.0)	78.5 (26.8)^a^

*BMI* body mass index, *FIM* functional independence measure, *IQR* interquartile range, *MMSE* mini mental state exam, *NRS* nutritional risk screening.

^a^Significant group difference (*p* <0.05) between the groups “acute geriatrics” and “geriatric rehabilitation”, as well as “males” and “females”.

### Prevalence of probable and confirmed sarcopenia

**Fig. 1:**
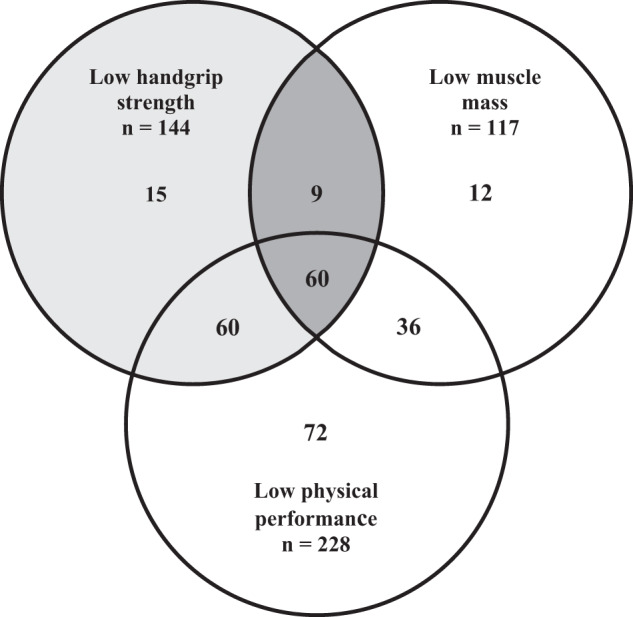
Assessment of sarcopenia in patients admitted to acute geriatrics and geriatric rehabilitation (*n* = 305). Probable sarcopenia was defined by low handgrip strength and normal muscle mass (*n* = 75; 24.6%). Sarcopenia was confirmed when both handgrip strength and muscle mass were low (*n* = 69; 22.6%), and was classified as severe by additional documentation of low physical performance (*n* = 60; 19.7%).

Results from the assessment of sarcopenia, stratified by wards and by gender, are summarized in Table 2 and Fig. 1. Out of 305 patients, 161 patients (52.8%; CI 47.2–58.4%) were classified as non-sarcopenic and 75 patients (24.6%; CI 19.8–29.4%) were classified as probable sarcopenic according to the criteria of EWGSOP2. Sarcopenia was diagnosed in 69 patients (22.6%; CI 17.9–27.3%), of which 60 patients (19.7%; CI 15.2–24.2%) fulfilled the criteria for severe sarcopenia. Patients admitted to acute geriatrics had a lower HGS than patients admitted to geriatric rehabilitation (39.0 kPa and 40.0 kPa, respectively, *p* = 0.048), and prevalence of probable sarcopenia was higher in acute geriatric patients (28.5% and 17.9%, respectively, *p* = 0.038). Compared to women, men showed a lower prevalence of probable sarcopenia (18.1% and 28.0%, respectively, *p* = 0.056) and a higher prevalence of confirmed sarcopenia (26.7% and 20.5%, respectively, *p* = 0.221), even though the differences were not found to be statistically significant. In the 37 patients assessed in acute geriatrics as well as geriatric rehabilitation, Cohen’s kappa coefficient of 0.778 showed substantial to high reliability for diagnosis of confirmed sarcopenia (Supplemental Table 1).

**Table 2:** Prevalence of sarcopenia, stratified by wards (acute geriatrics and geriatric rehabilitation) and by gender (males and females).

Characteristic	All *n* = 305	Acute geriatrics *n* = 193	Geriatric rehabilitation *n* = 112	Males *n* = 105	Females *n* = 200
Assessment of sarcopenia					
Handgrip strength, kPa, median (IQR)	39.0 (19.5)	39.0 (21.0)	40.0 (18.0)^a^	51.0 (20.0)	34.5 (14.8)^a^
ASMI, kg/m^2^, median (IQR)	6.2 (1.4)	6.2 (1.4)	6.4 (1.5)	7.0 (1.1)	5.8 (1.1)^a^
Low physical performance (TUG ≥20 s), *n* (%)	228 (74.8)	142 (73.6)	86 (76.8)	79 (75.2)	149 (74.5)
Prevalence of sarcopenia					
No sarcopenia, *n* (%)	161 (52.8)	92 (47.7)	69 (61.6)^a^	58 (55.2)	103 (51.5)
Probable sarcopenia, *n* (%)	75 (24.6)	55 (28.5)	20 (17.9)^a^	19 (18.1)	56 (28.0)
Confirmed sarcopenia, *n* (%)	69 (22.6)	46 (23.8)	23 (20.5)	28 (26.7)	41 (20.5)

*ASMI* appendicular skeletal muscle mass index, *IQR* interquartile range, *TUG* timed up and go test.

^a^Significant group difference (*p* <0.05) between the groups “acute geriatrics” and “geriatric rehabilitation”, as well as “males” and “females”.

### Parameters associated with probable and confirmed sarcopenia

Results of the multivariate regression analysis, adjusted for age and sex, to investigate parameters for association with probable and confirmed sarcopenia, are shown in Table 3. Low calf circumference (OR 4.05; CI 1.78–9.21), low BMI (OR 3.76; CI 1.59–8.88), cognitive impairment (OR 2.20; CI 1.01–4.81) and risk of malnutrition (OR 5.68; CI 2.28–14.13) were found to be positively associated with confirmed sarcopenia. In contrast, probable sarcopenia was found to be positively associated solely with cognitive impairment (OR 2.68; CI 1.36–5.28).

**Table 3:** Association of parameters with probable and confirmed sarcopenia.

Parameter	Association with probable sarcopenia^a^		Association with confirmed sarcopenia^b^	
	OR (95% CI)	*P* value	OR (95% CI)	*P* value
Low calf circumference (<31 cm)	1.52 (0.64–3.63)	0.348	4.05 (1.78–9.21)	*0.001*
Low mid-arm circumference^c^	1.29 (0.61–2.73)	0.501	0.64 (0.28–1.45)	0.284
Low BMI^d^	0.27 (0.08–0.89)	*0.031*	3.76 (1.59–8.88)	*0.003*
Cognitive impairment (MMSE <27 points)	2.68 (1.36–5.28)	*0.004*	2.20 (1.01–4.81)	*0.048*
Low physical performance (TUG ≥20 seconds)	1.44 (0.68–3.06)	0.339	2.25 (0.89–5.67)	0.086
At risk of malnutrition (NRS ≥3 points)	0.68 (0.35–1.29)	0.236	5.68 (2.28–14.13)	*<0.001*
Functional disability (FIM per 10 points decrease)	1.17 (0.99–1.39)	0.068	0.97 (0.81–1.17)	0.770

*BMI* body mass index, *CI* confidence interval, *FIM* functional independence measure, *MMSE* mini mental status exam, *NRS* nutritional risk screening, *OR* Odds ratio, *TUG* timed up and go test.

^a^Reference group: patients with no sarcopenia.

^b^Reference group: patients with no and probable sarcopenia.

^c^Cut-off points for low mid-arm circumference (MAC): <26.5 cm and <24.5 cm for men and women ≥75 years old, <27.5 cm and <25.5 cm for men and women <75 years old.

^d^Cut-off points for low BMI: <22 kg/m^2^ for persons ≥70 years old, <20 kg/m^2^ for persons <70 years old.

*P* values < 0.05 were considered to be statistically significant.

## Discussion

In this study, sarcopenia was diagnosed in 22.6% of geriatric inpatients, and multiple parameters were found to be independently associated with sarcopenia. To the best of the authors’ knowledge, this is the first study to investigate the prevalence of sarcopenia in both acute care and rehabilitation settings, and to evaluate parameters associated with probable and confirmed sarcopenia using diagnostic criteria of EWGSOP2.

Other studies reported on prevalence of sarcopenia in hospitalized older patients using criteria of EWGSOP2 and found prevalence rates of 18.1, 18.9 and 22.8% [16*–*18]. Although these data are similar to those found in our cohort, the participants of these three studies were younger than ours with a median age between 65.0 and 80.7 years. Furthermore, two of these studies used Dual Energy X-Ray Absorptiometry (DEXA) [16] or computed tomography [17] for quantification of muscle mass. However, numbers of comorbidities of our patients were comparable to those found in other studies [16, 18, 31]. In our cohort, baseline characteristics as well as prevalence of sarcopenia did not differ substantially between the two ward types, whereas probable sarcopenia (HGS below cut-off points) was detected significantly more often in acute geriatric patients than in patients of geriatric rehabilitation. This finding may be explained by the fact that patients of acute geriatrics presented a transiently aggravated muscle weakness due to acute illness, leading to a potential overestimation of probable and confirmed sarcopenia in acute patients. In contrast, patients admitted to geriatric rehabilitation might have benefitted from medical, nutritional and physiotherapy interventions during their preceding acute hospitalization. This may help to explain the lower prevalence of probable sarcopenia in patients of geriatric rehabilitation compared to acute geriatric patients.

Associations of low calf circumference [32], malnutrition [2] and cognitive impairment [3] with sarcopenia are well known. A previous pilot study used the new criteria of EWGSOP2 to analyze parameters for association with sarcopenia and found, in contrast to our data, only low MAC to be positively associated with the disease, although causality cannot be established in a cross-sectional study [33]. However, the sample size of this study was small (*n* = 40), and the patients were younger (mean age of 70.0 years) compared to our cohort. Our study confirms the association of calf circumference, low BMI, cognitive impairment and an increased risk of malnutrition with sarcopenia according to new criteria of EWGSOP2. We also demonstrate that all these parameters are correlated with confirmed sarcopenia, but not with probable sarcopenia. These findings suggest that assessment of both HGS and muscle mass are necessary to detect patients with low BMI and at risk for malnutrition who could benefit from nutritional and physiotherapy interventions. However, if assessment of muscle quantity, e.g., by using BIA or DEXA, is not available, clinical measures may support screening and evaluation of patients at risk for sarcopenia. These clinical measures including evaluation of nutritional status are easy available and part of the comprehensive geriatric assessment, which is routinely performed in geriatric hospitals [34].

Despite this fact, challenges in assessing geriatric inpatients also must be taken into account. Using EWGSOP2 cut-off points for low physical performance, a substantial proportion of both sarcopenic (87.0%) and non-sarcopenic (67.1%) participants of our cohort presented a TUG ≥ 20 s at admission. This finding is in line with other data demonstrating that in hospitalized geriatric patients with a mean age of 85.6 years, mean time to perform the TUG was 33.3 s [35]. Defined in 1991, the cut-off point of 20 s for the TUG was based on a community-dwelling population with a mean age of 79.5 years, in which all those who completed the TUG in <20 s were found to be independently mobile [24]. Compared to the elderly community-dwelling population, older hospitalized patients may have more mobility limitations and lower muscle strength due to acute illness [36]. Therefore, it is questionable whether the cut-off point of <20 s is adequate to define severe sarcopenia in older hospitalized patients. A universal definition of sarcopenia is still subject of ongoing debates [9], and additional data are needed to evaluate if a different cut-off point might be more appropriate to assess physical performance in geriatric hospitalized patients.

In this study, we were able to recruit 414 patients consecutively admitted to acute geriatrics and geriatric rehabilitation, with exclusion of only 72 patients due to exclusion criteria. Furthermore, we demonstrated that diagnostic measures for sarcopenia are feasible in geriatric hospital settings. The high Cohen’s kappa coefficient between the first and the second assessment in a subgroup of our study cohort reflects the high reliability of our data. Finally, we used a pneumatic hand dynamometer that is reliable and more practical compared to a hydraulic dynamometer to assess HGS in geriatric patients [37].

Some limitations need to be addressed. The results of this single-centre study focusing on Caucasian geriatric inpatients of a wealthy country are not generalizable. Also, as the highest measured BMI in this study was 35.0 kg/m^2^, our findings are not applicable for patients with a BMI >35 kg/m^2^. Therefore, multicentre-studies based on larger sample sizes are needed to enhance generalizability of the data. In our study, we used BIA for the evaluation of muscle mass, which relies on prediction equations to estimate different body compartments [38]. In older and obese patients, BIA potentially overestimates skeletal muscle mass compared to DEXA [39]. In addition, BIA results can also be influenced by changes in fluid distribution due to acute disease. Nevertheless, BIA is a portable and validated device that is harmless for the patient and is established for time-saving and cost-effective assessment of muscle mass [9, 39]. Furthermore, the geriatric baseline assessments were performed by various assessors in clinical daily practice with a potential to unequal application. However, assessment of HGS and muscle mass was performed by only two investigators. Finally, parts of the geriatric assessment included self-reported outcomes (e.g., NRS 2002 using self-reported weight loss or reduced food intake), which may be imprecise in patients with cognitive impairment. To reduce this bias, we excluded patients who were unable to follow the study procedure due to severe cognitive impairment, and only five (1.6%) of the included patients had a MMSE <10 points.

Despite these limitations, our study has clinical implications. To prevent further decline in sarcopenic geriatric patients, early diagnosis of this condition is crucial in order to timely target specific interventions designed to build up muscle strength and function. Therefore, in completion of the comprehensive geriatric assessment, it will be key to implement diagnostic measures for sarcopenia as a standard in daily practice to identify geriatric patients at risk. As our data demonstrate, the assessment of both muscle mass and function are needed to identify those vulnerable patients. Furthermore, specific treatments for sarcopenia, namely combined early nutritional and physiotherapy interventions, need to be further evaluated and implemented in order to delay disease progression and to improve clinical outcomes in sarcopenic patients.

## Conclusion

Sarcopenia is highly prevalent in patients admitted to acute geriatrics and geriatric rehabilitation, and multiple parameters were found to be associated with the disease. Routinely performed diagnostic measures at hospital admission and prompt interventional approaches are needed to build up muscle strength and function and to prevent adverse clinical outcomes in these patients.

## Supplementary information

Supplemental Table 1
