# Supplementary material for: Sarcopenia in hospitalized geriatric patients: insights into prevalence and associated parameters using new EWGSOP2 guidelines
Source: Eur J Clin Nutr. 2020 Oct 15;75(4):653–60. doi: 10.1038/s41430-020-00780-7 (PMC8035069; doi:10.1038/s41430-020-00780-7)
Supplement: Supplementary file 1 — Supplemental Table 1 [file 41430_2020_780_MOESM1_ESM.docx]

**Supplemental Table 1.** Characteristics of patients assessed in acute geriatrics as well as geriatric rehabilitation

| **Characteristic** | **Acute geriatrics**  **n = 37** | **Geriatric rehabilitation**  **n = 37** |
| --- | --- | --- |
| ***General characteristics*** | | |
| Age, years, median (IQR) | 85.0 (11.0) | |
| BMI, kg/m^2^, median (IQR) | 26.6 (7.6) | 26.8 (7.5) |
| Calf circumference, cm, median (IQR) | 32.5 (5.3) | 32.5 (4.3) |
| Mid-arm circumference, cm, median (IQR) | 25.0 (5.8) | 25.0 (5.8) |
| Phase angle, °, median (IQR) | 4.3 (0.9) | 4.3 (0.9) |
| Bedridden, n (%) | 18 (48.6) | 5 (13.5)^a^ |
| Length of hospital stay, days, median (IQR) | 18.0 (7.0) | 19.0 (7.0) |
| ***Geriatric assessment parameters*** | | |
| Cognitive Impairment (MMSE <27 points), n (%) | 25 (67.6) | 24 (64.9) |
| At risk of malnutrition (NRS ≥3 points), n (%) | 23 (62.2) | 24 (64.9) |
| FIM score, points, median (IQR) | 69.0 (24.0) | 85.0 (20.0)^a^ |
| ***Assessment of sarcopenia*** | | |
| Handgrip strength, kPa, median (IQR) | 33.0 (21.5) | 36.0 (19.0) |
| ASMI, kg/m^2^, median (IQR) | 6.0 (1.3) | 6.0 (1.2) |
| Low physical performance (TUG ≥ 20 seconds), n (%) | 33 (89.2) | 29 (78.4) |
| ***Prevalence of sarcopenia*** | | |
| No sarcopenia, n (%) | 17 (46.0) | 19 (51.4) |
| Probable sarcopenia, n (%) | 10 (27.0) | 9 (24.3) |
| Confirmed sarcopenia, n (%) | 10 (27.0) | 9 (24.3) |
| Cohen’s kappa coefficient for confirmed sarcopenia | 0.788 | |

^a^ Significant group difference (*p* <0.05) between the groups “acute geriatrics” and “geriatric rehabilitation”.

Abbreviations: ASMI, appendicular skeletal muscle mass index; BMI, body mass index; FIM, functional independence measure; IQR, interquartile range; MMSE, mini mental state exam; NRS, nutritional risk score; TUG, timed up and go test.
